# Editorial: Advances in veterinary tissue engineering: unlocking potential with cell-free and cell-based methods

**DOI:** 10.3389/fvets.2025.1591272

**Published:** 2025-03-26

**Authors:** Khan Sharun, Hussein M. El-Husseiny, Sathish Muthu

**Affiliations:** ^1^Center for Regenerative Nanomedicine, Northwestern University, Chicago, IL, United States; ^2^Graduate Institute of Medicine, Yuan Ze University, Taoyuan, Taiwan; ^3^Institute of Global Innovation Research, Tokyo University of Agriculture and Technology, Fuchu, Japan; ^4^Department of Surgery, Anesthesiology, and Radiology, Faculty of Veterinary Medicine, Benha University, Toukh, Egypt; ^5^Laboratory of Veterinary Physiology, Department of Veterinary Medicine, Tokyo University of Agriculture and Technology, Tokyo, Japan; ^6^Department of Orthopaedics, Orthopaedic Research Group, Coimbatore, Tamil Nadu, India; ^7^Central Research Laboratory, Meenakshi Medical College Hospital and Research Institute, Meenakshi Academy of Higher Education and Research, Chennai, India

**Keywords:** platelet-rich plasma, stem cells, wound regeneration, extracellular vesicles, bone regeneration, veterinary regenerative medicine

## Introduction

Tissue engineering has emerged as a transformative field in veterinary medicine, offering innovative solutions for tissue repair, regeneration, and replacement (1). As traditional therapeutic approaches often fail to achieve complete tissue repair or functional restoration, the field has increasingly turned to interdisciplinary strategies that merge tissue engineering, stem cell biology, and biomaterial science (2, 3). These innovations not only aim to address the limitations of conventional treatments but also emphasize sustainability, scalability, and translational potential. With the growing demand for advanced therapeutic strategies to address complex injuries, degenerative diseases, and congenital anomalies in animals, the integration of cell-free and cell-based methods has opened new avenues for research and clinical application (4, 5). This Research Topic serves as a testament to the dedication and ingenuity of researchers who are pushing the boundaries of this exciting field, paving the way for a future where tissue regeneration is a reality for all animals.

A key theme emerging from the papers published in this Research Topic is the sustainable use of biological resources to develop cost-effective and biocompatible materials for clinical applications (6). From repurposing seafood by-products to engineering scaffolds for periodontal regeneration, researchers are pushing the boundaries of innovation while embracing principles of circular economy and environmental responsibility. The use of stem cells, whether derived from the periodontal ligament, deer antlers, or human embryonic sources, further highlights the growing potential of cell-based and cell-free therapies in veterinary regenerative medicine (2). Additionally, clinical investigations into biomaterial-assisted bone healing and extracellular vesicle therapy in aging dogs underscore the translational impact of these emerging technologies.

One of the promising studies in this Research Topic is written by Zivelonghi et al. that explores the potential of seafood by-products in tissue engineering. This study ingeniously repurposes sea urchin food waste to create collagen-based wound dressings. By leveraging the principles of circular economy and blue biotechnology, the authors demonstrate the feasibility of extracting valuable biomaterials from readily available by-products. The ex vivo wound healing organ culture model, utilizing rat skin explants, effectively assessed the regenerative potential of these dressings. Notably, the antioxidant-enriched marine collagen wound dressing showed promising results in accelerating skin wound healing by mitigating inflammation. This work highlights the potential of marine-derived biomaterials for developing cost-effective and environmentally friendly wound healing solutions, offering a compelling alternative to traditional collagen-based dressings.

Periodontal disease, including gingivitis and periodontitis, is a prevalent oral condition caused by dental plaque invasion, leading to the destruction of periodontal tissues (7). While conventional treatments can reduce inflammation and remove plaque, they often fail to achieve complete regeneration of the complex periodontal structure. Recognizing the limitations of traditional treatments, the study by Dai et al. establishes a protocol for isolating canine periodontal ligament stem cells (PDLSCs) and fabricates a biocompatible scaffold using chitosan, β-glycerol phosphate, and biphasic calcium phosphate. Co-culturing PDLSCs with the scaffold creates a cell-scaffold complex with demonstrated *in vitro* and *in vivo* biocompatibility, showing no inflammatory reactions when transplanted into canine mandibular molar defects. This research highlights the potential of PDLSC-seeded tissue engineering scaffolds to repair periodontal defects and enhance guided tissue regeneration, offering a significant advancement over conventional approaches.

Deer antlers exhibit a unique regenerative ability, making them a valuable model for studying tissue engineering. Chen et al. explored the potential of deer antler stem cells (AnSCs) for regenerative medicine. The remarkable regenerative capabilities of deer antlers have long fascinated scientists, and this study delves into the mechanisms underlying this phenomenon. By transducing the human telomerase reverse transcriptase (hTERT) gene, the authors successfully immortalized AnSCs, creating a cell line with extended proliferative capacity while maintaining stem cell characteristics. The derived small extracellular vesicles (sEVs) also exhibited desirable properties, mirroring those of primary AnSCs. This work provides a valuable resource for researchers seeking to harness the regenerative potential of AnSCs and their sEVs. The ability to generate a stable and scalable cell line opens up new avenues for exploring the therapeutic applications of these cells in various regenerative medicine contexts.

Bone regeneration remains a major focus in veterinary orthopedics, particularly for managing complex fractures. The case report by Barbaro et al. presents a novel approach using a combination of platelet-rich plasma (PRP) and hydroxyapatite nanoparticles to accelerate bone healing in a Rottweiler dog with a severe tibial fracture. Traditional surgical intervention failed to yield satisfactory results, but the application of PRP and hydroxyapatite demonstrated significant improvements in bone regeneration within 10 days post-treatment. Radiographic assessments showed increased callus formation and reduced fracture gaps, highlighting the potential of biomaterial-assisted regenerative therapy. This study provides valuable insights into the clinical application of PRP and hydroxyapatite in veterinary orthopedics, offering a promising treatment option for severe bone injuries.

Another important clinical study by Kim et al. evaluated the therapeutic potential of human embryonic stem cell-derived mesenchymal stem cells (ES-MSCs) and their extracellular vesicles (ES-MSC-EVs) in improving cognitive and mobility function in geriatric dogs. The study assessed cognitive and behavioral changes using the Canine Cognitive Dysfunction Rating (CCDR) and mobility impairment through the Liverpool Osteoarthritis in Dogs (LOAD) scale before and two weeks after treatment. Notably, both ES-MSC and ES-MSC-EV treatments led to significant improvements in CCDR and LOAD scores, with no reported adverse effects based on comprehensive blood tests. These findings highlight the promise of cell-free regenerative strategies, particularly extracellular vesicle-based therapies, in addressing age-related neurodegenerative and musculoskeletal disorders in dogs.

Collectively, these five papers showcase the breadth and depth of research within veterinary tissue engineering. From sustainable biomaterials derived from seafood waste to gene-edited stem cells for allogeneic transplantation and from immortalized deer antler stem cells to combined therapies for bone regeneration and cell-free regenerative strategies for improving cognitive and mobility function in geriatric dogs, these studies exemplify the innovative approaches being explored to address diverse clinical needs. By embracing multidisciplinary collaboration and pushing the boundaries of scientific discovery, we can continue to unlock the full potential of veterinary regenerative medicine.

## Summary

Advances in veterinary tissue engineering are revolutionizing how we approach animal tissue repair and regeneration. By unlocking the potential of cell-free and cell-based methods, researchers and clinicians can develop innovative solutions that address the unique challenges of veterinary medicine. The synergistic integration of these approaches offers a promising pathway for achieving functional and structural regeneration, improving the quality of life for animals, and advancing the field of veterinary regenerative medicine. As we continue to explore new frontiers in tissue engineering, collaboration among researchers, clinicians, and industry stakeholders will be essential to translate these advancements into practical and accessible therapies for veterinary patients.
